# Supplementary material for: High-Throughput 16S rRNA Sequencing to Assess Potentially Active Bacteria and Foodborne Pathogens: A Case Example in Ready-to-Eat Food
Source: Foods. 2019 Oct 11;8(10):480. doi: 10.3390/foods8100480 (PMC6836182; doi:10.3390/foods8100480)
Supplement: Supplementary file 1 [file foods-08-00480-s001.pdf]

Table S1. Relative abundance of different classified bacterial genera.

	S1 (RNA)	S2 (RNA)	S3 (RNA)	S1 (DNA)	S2 (DNA)	S3 (DNA)
Pseudomonas	53.3	65.1	67.8	60.9	77.8	75.5
Serratia	4.9	0.7	0.2	9.4	0.5	0.1
Janthinobacterium	1.5	0.2	0.010	4.7	0.5	0.3
Flavobacterium	1.4	0.4	0.1	11.4	3.4	0.7
Rahnella	1.4	5.4	4.2	2.8	7.9	4.2
Pantoea	0.1	2.0	3.1	0.2	3.4	6.1
Other genera (<1% of abundance each)	0.5	0.5	1.8	1.9	1.0	5.7
Unclassified at the genus level	36.9	25.6	22.8	8.7	5.4	7.5

Table 2. Relative abundance of genera detected in analyzed RTE salad brands (DNA- and RNA-based approaches).

	RNA			DNA		
**Genus**	**S1 (RNA)**	**S2 (RNA)**	**S3 (RNA)**	**S1 (DNA)**	**S2 (DNA)**	**S3 (DNA)**
Acidaminobacter	0	0	0	0	0	0.00414662
Acinetobacter	0	0.00896392	0.07510327	0.00209565	0.03626827	0.46856859
Aerococcus	0.00126165	0	0	0	0	0
Aeromonas	0	0	0.03359883	0	0	0.07049262
Alkanindiges	0	0	0.00790561	0	0.00278987	0.07049262
Allorhizobium	0.00252331	0.00298797	0.01185841	0.01886081	0.0167392	0.09122574
Aquipuribacter	0	0	0.0019764	0	0	0
Arcobacter	0	0	0	0	0	0.00829325
Arthrobacter	0	0	0	0	0	0.01243987
Bacillus	0	0.00149399	0.02964603	0	0.0083696	0.08293249
Bosea	0	0	0	0	0	0.00414662
Bradyrhizobium	0	0	0	0	0	0.00414662
Brevundimonas	0	0.00448196	0.01778762	0	0	0.033173
Buchnera	0	0	0	0	0	0.02487975
Burkholderia	0	0	0	0	0.00278987	0
Caulobacter	0	0	0	0	0	0.00829325
Cavicella	0	0	0	0	0	0.00414662
Chryseobacterium	0.00126165	0.00298797	0.01185841	0.00419129	0.04184801	0.16171836
Clostridium	0	0	0	0	0.00557973	0.00414662
Conexibacter	0	0	0.0039528	0	0	0
Curtobacterium	0.00504662	0.11802495	0.93879084	0	0.03626827	0.21562448
Cutibacterium	0	0	0	0.01047823	0	0
Devosia	0	0	0.0039528	0	0	0
Domibacillus	0	0	0	0	0	0.00414662
Duganella	0.02397144	0.07320535	0.0039528	0.15088646	0.20645017	0.07463924
Dyadobacter	0.00126165	0.00149399	0	0.01886081	0.0083696	0.00414662
Empedobacter	0	0	0	0.00209565	0	0
Enterobacter	0	0.00149399	0.02766962	0.00628694	0.00278987	0.0497595
Flavobacterium	1.4	0.4	0.1	11.4	3.4	0.7
Hafnia-Obesumbacterium	0	0	0.00592921	0	0	0.00414662
Herbaspirillum	0	0	0	0	0	0.02073312
Herminiimonas	0.00126165	0	0	0.00838258	0.00278987	0
Hydrogenophaga	0	0	0	0	0	0.00414662
Iamia	0	0	0.0019764	0	0	0
Janthinobacterium	1.484	0.191	0.010	4.665	0.513	0.332
Kaistia	0	0	0	0	0	0.00414662
Kosakonia	0	0	0.01185841	0	0	0.01243987

Lachnospiraceae NK4A136 group	0.00126165	0	0	0	0.00278987	0
Lachnospiraceae UCG-006	0	0.00149399	0	0	0	0
Lactococcus	0.00126165	0	0.02174042	0.00838258	0.05300748	0.27782385
Leuconostoc	0	0	0.0019764	0.01047823	0.08369602	0.02487975
Luteibacter	0.00252331	0.00448196	0.00988201	0.00209565	0.00557973	0
Luteolibacter	0	0	0	0.05239113	0	0
Massilia	0.00504662	0.00448196	0.0039528	0	0.01115947	0.05805275
Methylobacterium	0.00126165	0	0.0019764	0	0.0083696	0.033173
Methylophaga	0	0	0	0	0	0.00414662
Methylotenera	0	0	0	0.01047823	0.02231894	0.02073312
Microbacterium	0	0	0.0039528	0	0	0.02073312
Microvirga	0	0	0	0	0	0.00414662
Mucilaginibacter	0.01261655	0	0	0.05239113	0.00278987	0.00829325
MWH-UniP1 aquatic group (Burkholderiaceae)	0	0	0.0019764	0	0	0
Nocardioides	0	0	0.0019764	0	0	0.00414662
Novosphingobium	0.00126165	0	0.0019764	0.00628694	0.00278987	0.0165865
Oscillibacter	0.00252331	0	0	0	0	0
Paenarthrobacter	0	0	0	0	0	0.00414662
Pantoea	0.1	2.0	3.1	0.2	3.4	6.1
Parafrigoribacterium	0.00126165	0	0	0	0	0
Patulibacter	0	0	0	0.00209565	0	0
Pectobacterium	0	0	0.0039528	0	0	0.00414662
Pedobacter	0.29270385	0.02091581	0.01581121	1.435517	0.15623256	0.32758335
Pigmentiphaga	0.00126165	0	0	0	0	0
Pseudarthrobacter	0	0	0.00988201	0	0	0
Pseudofulvibacter	0	0	0.0019764	0	0	0
Pseudomonas	53.2922875	65.1497722	67.8241793	60.9476508	77.8093963	75.4519821
Pseudonocardia	0	0	0	0	0	0.00414662
Pseudorhodoferax	0	0.00149399	0	0	0	0
Psychrobacter	0	0	0.0039528	0	0	0.00829325
Rahnella	1.4	5.4	4.2	2.8	7.9	4.2
Rheinheimera	0.00126165	0.00597595	0.00790561	0.00209565	0.03905814	0.033173
Rhodanobacter	0	0	0.0039528	0	0	0.00414662
Rhodococcus	0	0.00149399	0.0019764	0	0.00278987	0.00829325
Rhodoferax	0.00252331	0	0	0	0.00278987	0
Sanguibacter	0	0	0.01581121	0	0	0
Serratia	4.9	0.7	0.2	9.4	0.5	0.1
Shewanella	0	0	0.31029508	0	0.00278987	3.06020899
Sphingobacterium	0.01009324	0	0	0.064965	0	0.00829325
Sphingobium	0.00504662	0.02539777	0.0039528	0.00628694	0.03626827	0
Sphingomonas	0.0277564	0.20467618	0.15613574	0.01886081	0.22876911	0.21147786
Stenotrophomonas	0.01261655	0.00896392	0.01383481	0.01676516	0.00557973	0.0497595
Streptococcus	0	0	0.0019764	0	0	0
Streptomyces	0	0	0	0	0	0.00414662
Unclassified at genus level	36.9122267	25.6487637	22.825464	8.65291923	5.38165383	7.49295074
Variovorax	0.05929776	0.00149399	0.00988201	0.00838258	0	0.00414662
Verticia	0.00126165	0	0	0	0	0
Xanthomonas	0	0.00149399	0.0019764	0	0.00557973	0
Yersinia	0	0.00149399	0	0	0.00278987	0
TOTAL	100	100	100	100	100	100
